# Short-Term Memory Deficits in the SLEEP Inbred Panel

**DOI:** 10.3390/clockssleep1040036

**Published:** 2019-10-28

**Authors:** Shailesh Kumar, Kirklin R. Smith, Yazmin L. Serrano Negron, Susan T. Harbison

**Affiliations:** Laboratory of Systems Genetics, National Heart Lung and Blood Institute, Bethesda, MD 20892, USA; shailesh.kumar2@nih.gov (S.K.); yazmin.serranonegron@nih.gov (Y.L.S.N.)

**Keywords:** *Drosophila melanogaster*, sleep, circadian rhythms, learning and memory

## Abstract

Although sleep is heritable and conserved across species, sleep duration varies from individual to individual. A shared genetic architecture between sleep duration and other evolutionarily important traits could explain this variability. Learning and memory are critical traits sharing a genetic architecture with sleep. We wanted to know whether learning and memory would be altered in extreme long or short sleepers. We therefore assessed the short-term learning and memory ability of flies from the Sleep Inbred Panel (SIP), a collection of 39 extreme long- and short-sleeping inbred lines of *Drosophila*. Neither long nor short sleepers had appreciable learning, in contrast to a moderate-sleeping control. We also examined the response of long and short sleepers to enriched social conditions, a paradigm previously shown to induce morphological changes in the brain. While moderate-sleeping control flies had increased daytime sleep and quantifiable increases in brain structures under enriched social conditions, flies of the Sleep Inbred Panel did not display these changes. The SIP thus emerges as an important model for the relationship between sleep and learning and memory.

## 1. Introduction

Sleep is a highly conserved behavior [1,2,3,4,5]. Despite this conservation, considerable variation in sleep duration exists among species [5,6,7]. Variability in sleep duration has also been documented within a species; specifically, it has been observed in humans, mice, and *Drosophila* [8,9,10,11,12]. Though it has a clear genetic component, the underlying reasons for variation in sleep duration remain unknown [10,13,14,15,16,17]. To facilitate the study of this variability, we created a panel of 39 wild-derived inbred lines with extremely long and short sleep duration, the Sleep Inbred Panel (SIP) [18]. The SIP is a useful tool to study differential transcript and protein abundances between long and short sleepers; to verify polymorphic variants implicated in genome-wide association studies of sleep duration; and to identify important phenotypic correlates of sleep [18]. 

One potential correlate of interest is the ability to acquire knowledge and retrieve it—learning and memory. In fact, a role in learning and consolidation of memory have long been proposed as a potential function of sleep [19]. In addition to being a model for sleep [20,21], *Drosophila* have been established as a model for learning, short-term memory, and long-term memory. Several paradigms exist to test learning and short-term memory in flies. The aversive phototaxis suppression assay is a short-term memory paradigm that takes advantage of a fly’s natural inclination to move towards a light source [22,23]. If given a choice between a lighted and a dark chamber, the fly would prefer the lighted one. However, the fly can be trained to avoid a lighted chamber in favor of a dark one if an aversive stimulus, such as quinine, is added to the lighted chamber [22,23]. Short-term memory can also be tested by exposing a fly to a heat source [24]. A wildtype fly is naturally repelled by high temperatures; given a chamber with a high temperature side (37 °C) and a low temperature side (25 °C), the fly will choose the low temperature side [25]. If flies are repeatedly exposed to the two-temperature chamber, they can be trained to move to a specific side [24]. Long-term memory can also be tested in flies. Olfaction is one way to test long-term memory. Two aversive odors are presented to a group of flies in a T-maze. One of the odors is coupled with an electric shock. After several trials, the flies learn to choose the odor that is not associated with the shock, and they retain the memory for 24 h [26]. Long-term memory can also be tested through the courtship paradigm [27]. Virgin male flies readily court virgin females; however, if a virgin male tries to court a mated female, she will reject him. The male will then be slower to court the next female he encounters. The memory of this experience can last up to 9 days [27]. These paradigms have been used to explore the relationship between sleep and short- and long-term memory in flies.

Several lines of evidence have established a relationship between sleep and learning and memory in flies. First, sleep deprivation has a negative effect on learning and memory in wildtype flies. Sleep deprivation for as little as 6 h prior to training disrupts aversive phototaxis memory [28]. Short-term olfactory memory retention was also impaired in flies after 24 h of sleep deprivation [29]. Long-term memories are sensitive to sleep deprivation that occurs after training. If flies were deprived of 4 h of sleep immediately after training, they had defective courtship memory [30]. Thus, both short-term and long-term learning and memory can be affected by sleep loss in wildtype flies.

Second, some genes that impact sleep duration also have learning and memory defects, suggesting a shared genetic architecture between these traits. Loss-of-function mutations and knockdowns in genes such as *fumin*, *CanA-14F*, *crossveinless-c*, *highwire*, *Hyperkinetic*, *Resistant to dieldrin*, and *Shaker* reduce sleep duration [31,32,33,34,35,36]. *fumin* mutants have defects in aversive olfactory retention [37] as do *CanA-14F* mutants [34]. Testing different mutant alleles of *Hyperkinetic* and *Shaker* revealed that only alleles that result in short sleep have reduced short-term memory retention in the heat box assay [33], demonstrating the genetic link between the two traits. *crossveinless-c* mutants had olfactory memory impairments [36]. Additionally, a reduction in *Resistant to dieldrin* expression in both mushroom body and clock neurons protects flies against performance deficiencies in the aversive phototaxis assay [35]. Interestingly, increasing sleep pharmacologically and genetically restored both short-term and long-term memory in *dunce* and *rutabaga* mutants [38].

In addition to the learning and memory paradigms listed above, it was recently proposed that social enrichment in flies could be used as a marker for synaptic plasticity. Social experience involves genes that are important for learning and memory as well as synaptic functions and plasticity [39,40,41]. Social enrichment has been shown to enhance cognitive and behavioral performance in mammals [42,43] and affect subsequent sleep in flies [30]. Wildtype flies exhibit a robust increase in day sleep when they are exposed to socially enriched environments, such as being housed at 30–45 flies in a same-sex vial [30]. Flies housed in this way will increase their day sleep relative to individually-housed flies [30]. These effects last for at least three days [44], but the flies’ response to social enrichment declines with age [45]. Behavioral effects of social exposure are accompanied by structural changes in the brains of the flies. Specifically, the numbers of PDF-positive punctae in ventral lateral neuronal projections in the accessory medulla increase under conditions of social exposure [46]. Social exposure also increased branches in the giant tangential neuron of the lobula plate vertical system (VS1) [47]. Thus, the social exposure paradigm can be used to identify changes in neural plasticity.

Here we tested long and short sleepers of the SIP for differences in learning and memory. We used the aversive phototaxis assay to measure short-term memory in the SIP and compared the results to a moderate-sleeping control line. We also tested flies for their behavioral and morphological responses to social enrichment. Both long and short sleepers were unexpectedly deficient in their ability to acquire short-term memories and in their response to social enrichment.

## 2. Results

### 2.1. Aversive Phototaxis Assay Performance in the SIP

We used the Aversive Phototaxis Suppression assay to test short-term learning and memory in long and short sleeping flies of the SIP [22,23]. With this paradigm, a fly is given a choice between two chambers: a lighted chamber and a darkened chamber. Flies are naturally phototactic and are attracted to the lighted chamber. However, they can be trained to choose the darkened chamber if they are provided with an aversive stimulus, namely filter paper soaked with quinine, in the lighted chamber. Flies are tested in four blocks of four trials; the number of times the fly chooses the dark chamber in the last block of four trials is its performance score on the assay.

We compared scores in long and short sleeping lines to that of a moderate-sleeping line as a control. We used DGRP_373 from the Drosophila Genetic Reference Panel (DGRP), a group of wild-derived inbred lines, as a control [48,49]. Mean night sleep for the entire DGRP was 552.2 ± 6.73 min, while the median was 568.9 min [10]. Mean day sleep for the entire DGRP was 370.5 ± 7.25 min, while the median was 369.8 min [10]. Of the 167 lines measured, DGRP_373 had day and night sleep duration that was closest to both the mean and the median sleep of the DGRP. The mean night sleep for DGRP_373 was 567.9 ± 12.3 min, while the mean day sleep was 364.4 ± 17.1 min. Sleep in DGRP_373 therefore represents moderate sleep.

**Figure 1:**
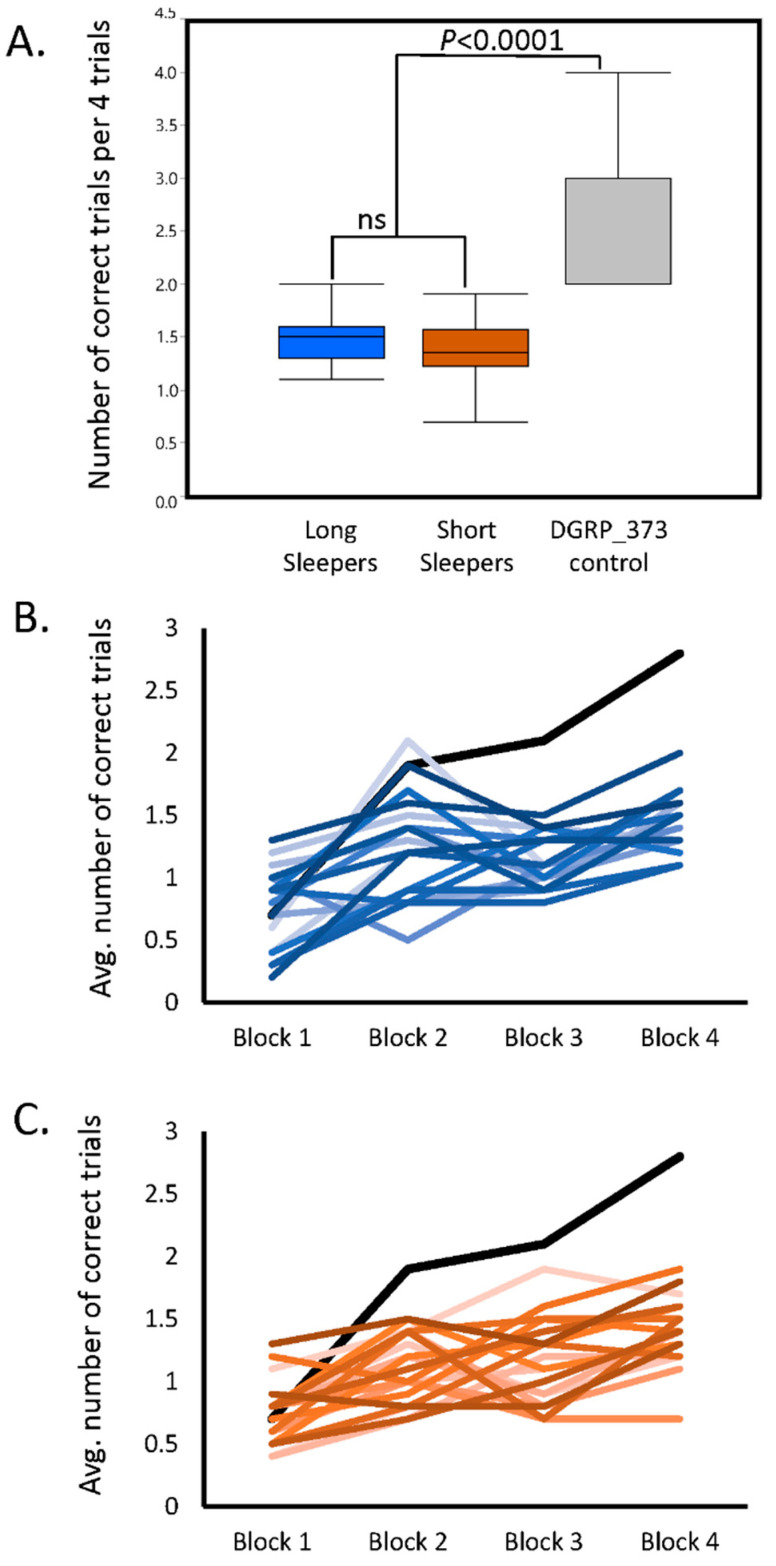
Deficient short-term learning in the Sleep Inbred Panel. (**a**) Comparison of Block 4 average performance scores in long and short sleeping lines to the moderate-sleeping DGRP_373 control; (**b**) Comparison of average performance scores across all four test blocks for long sleeping lines of the SIP (blue lines) with DGRP_373 (black line); (**c**) Comparison of average performance scores across all four test blocks for short sleeping lines of the SIP (orange lines) with DGRP_373 (black line).

Compared to the DGRP_373 control, both the long and short sleepers of the SIP had very low average scores on the assay (one-way analysis of variance *p* < 0.0001; Figure 1A; see Table S1 for the average score per SIP line). Scores ranged from 1.0 to 2.0 for long sleepers, and from 0.7 to 1.9 for short sleepers. Interestingly, the average of all long sleeper scores was not significantly different from the average of all short sleeper scores. In fact, both long and short sleepers appeared to have profound short-term memory defects.

The poor performance may be due to a defect in short-term memory, or it may be a defect in the rate of acquisition [23]. To determine whether the low scores were due to a defect in acquisition, we compared the performance scores of Block 1 to the performance scores of Block 4 for each line (i.e., the approximate slope of the learning curve). Sixteen SIP lines showed no improvement in their memory after repeated trials. The remaining 23 SIP lines plus the DGRP_373 control had performance scores in Block 4 that were significantly greater than Block 1 (two-way analysis of variance *p* < 0.05), indicating some degree of improvement in performance. Three lines: SIP_L1_4, SIP_L2_2, and SIP_S1_5 had significant Block × Sex interactions (*p* < 0.05), indicating that males and females of these lines responded differently to training. The improvement in score across all four blocks in the long and short sleepers can be seen in Figure 1B and 1C, with the DGRP_373 control plotted in each figure for comparison. While some long and short sleepers show minor improvements in score over the four trials, they never reached the level of performance observed in DGRP_373. Thus, the short and long sleepers both had deficits in memory acquisition.

We calculated the correlation between day and night sleep duration and performance score for socially isolated and enriched sleep (Figure S1). For short sleepers, we observed a significant negative correlation between performance score and day sleep under isolated conditions, and night sleep was negatively correlated with performance score for both social conditions. Performance score and sleep were not significantly correlated in long sleepers. The relationship between sleep duration and performance score was not linear, therefore, across the full range of day or night sleep.

### 2.2. Proboscis Extension Reflex Response to Quinine

**Figure 2:**
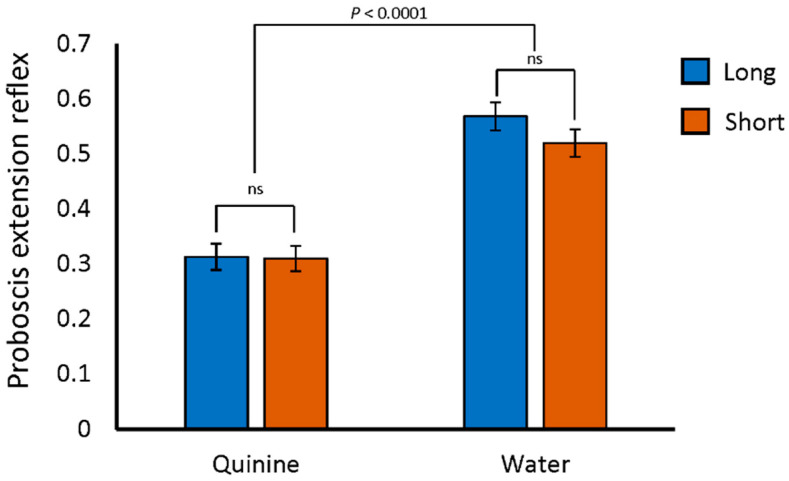
Normal aversive response to quinine in the SIP. Average proportion of long and short sleeper lines responding to water and quinine stimuli. Neither long nor short sleeper responses were significantly different from DGRP_373 responses to water (0.55; *p* = ns by post-hoc Dunnett) nor quinine (0.45; *p* = ns by post-hoc Dunnett).

One potential reason for reduced performance on the aversive phototaxis assay would be an inability to detect quinine, the negative reinforcement stimulus. In fact, *Gr33A*, a gustatory receptor required for the avoidance of bitter tastants [50], was previously identified as a gene differentiating extreme long and short sleep [51]. Flies of the SIP are polymorphic for a variant located 573 bp upstream of *Gr33A* [18]. All of the long sleepers in the SIP are homozygous for the “G” allele at this variant, which is the same as the reference *Drosophila* sequence. The short sleepers, however, are homozygous for the alternate “C” allele at this position. If either of these two variants affect *Gr33A*, the flies may not be able to detect bitter stimuli. We therefore tested the flies for their avoidance of quinine using the proboscis extension reflex assay [52]. With this assay, flies are exposed to both quinine and a water control after being held in vials with no food or water for 1.5 h. The legs of each fly, which have taste receptors, were exposed to a drop of water or quinine for 5 s. If the fly responded with a proboscis extension, it was noted. We found no differences among the percentages of long sleepers, short sleepers, or the DGRP_373 control responding to the water stimulus with proboscis extension. Nor did we detect any difference in proboscis extension reflex between long sleepers, short sleepers, or DGRP_373 when quinine was used as the stimulus. Flies of all genotypes favored the water over the quinine stimulus (Figure 2 and Table S1, *p* < 0.0001 by two-way ANOVA). Thus, both long and short sleepers were able to detect and respond to the bitter quinine stimulus.

### 2.3. Sleep in the SIP under Isolated and Socially Enriched Conditions

**Figure 3:**
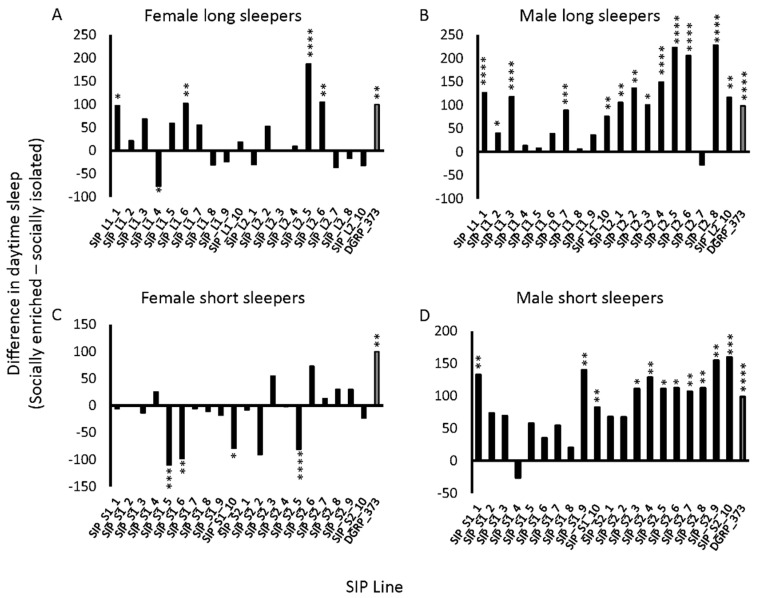
Variable response to social enrichment in the SIP. The difference in average day sleep under socially enriched and isolated conditions is plotted for (**a**) female long sleepers; (**b**) male long sleepers; (**c**) female short sleepers; and (**d**) male short sleepers. DGRP_373 is plotted as gray bars. * 0.01 ≤ *p* < 0.05; ** 0.001 ≤ *p* < 0.01; *** 0.0001 ≤ *p* < 0.001; **** *p* < 0.0001.

Flies exposed to enriched social conditions increase their daytime sleep relative to isolated flies for several days following the enrichment [30,46]. This increase in daytime sleep was previously proposed as an indicator of neuronal plasticity [45]. We hypothesized that because the SIP did not perform well on the Aversive Phototaxis Assay, they might not respond to social exposure as expected. We subjected male and female flies of the SIP to the social exposure paradigm (Methods) and measured their sleep afterwards. Day sleep in the SIP was both increased and decreased under social enrichment relative to social isolation (Figure 3; Table S2). Females had fewer increases in daytime sleep with social enrichment, and in some cases, there were significant decreases in daytime sleep with enrichment, particularly in the short sleepers (Figure 3A,C). Thirty of the SIP lines had no response to social enrichment in females. Of these 30 lines, six had increases in night sleep or average night bout length, and four had increases in average day bout length, suggesting increased consolidation of sleep with social exposure (Table S3) [45]. Males had more robust increases in day sleep than females, whether the flies were short sleepers or long sleepers (Figure 3B,D). While 15 of the SIP lines had no response to social enrichment in males, 7 lines had increases in sleep duration or average bout length suggesting increased consolidation (Table S3). Overall, the panel’s response to enriched social conditions varied and was stratified by both genotype and sex.

### 2.4. PDF Release Site Morphology Changes in SIP Representative Lines under Isolated and Socially Enriched Conditions

**Figure 4:**
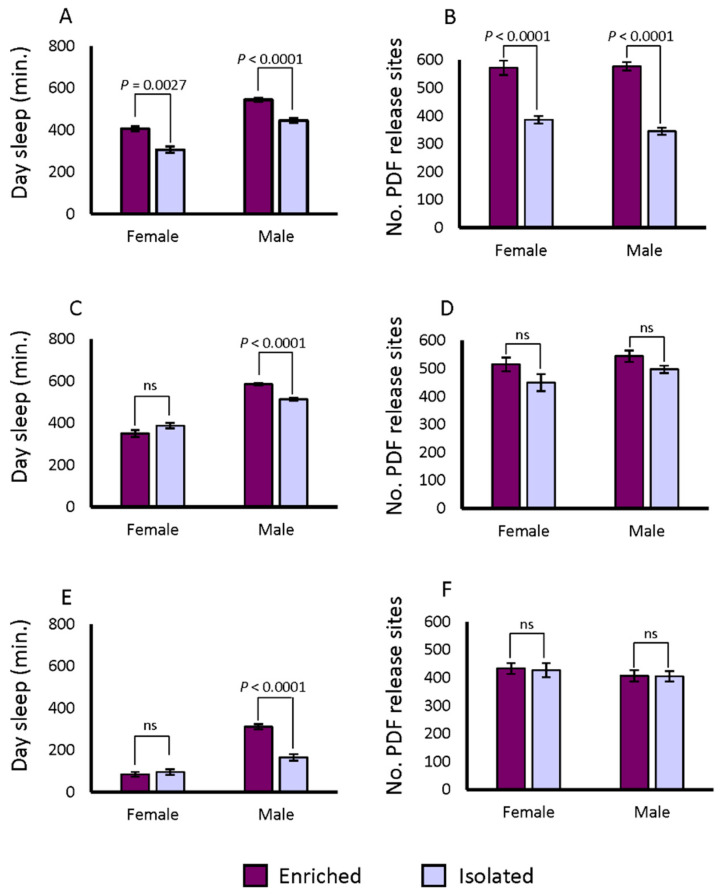
Changes in day sleep and numbers of PDF release sites after social exposure. The difference in average day sleep under socially enriched and isolated conditions is plotted for (**a**) DGRP_373, (**c**) SIP_L1_1, and (**e**) SIP_S2_7. Differences in PDF release sites under isolation and social enrichment for (**b**) DGRP_373, (**d**) SIP_L1_1, and (**f**) SIP_S2_7. * 0.01 ≤ *p* < 0.05; ** 0.001 ≤ *p* < 0.01; *** 0.0001 ≤ *p* < 0.001; **** *p* < 0.0001.

**Figure 5:**
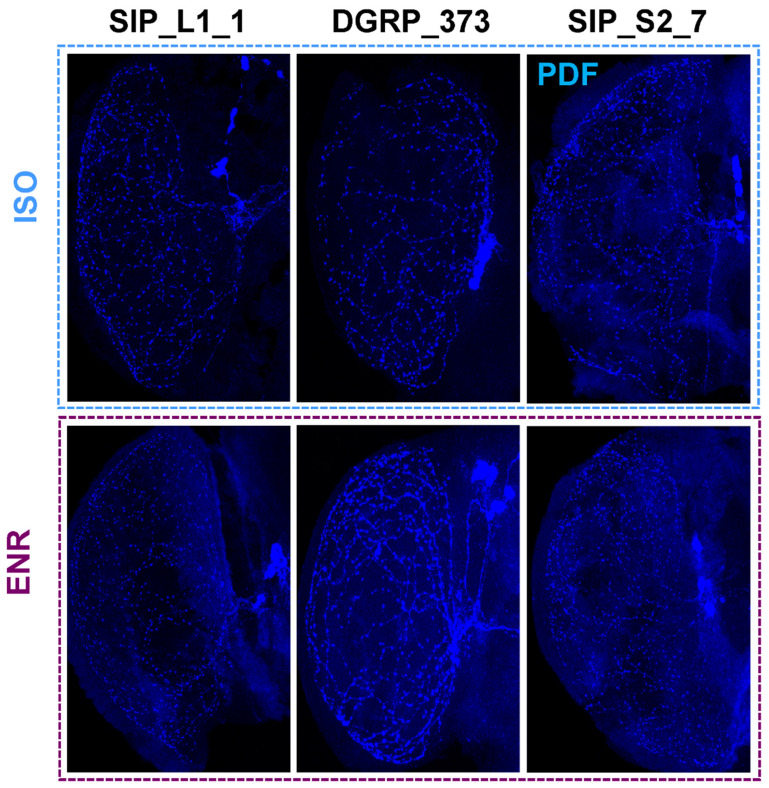
Representative PDF release site staining in the medulla. PDF-stained medulla for SIP_L1_1, DGRP_373, and SIP_S2_7 are shown. ISO isolated flies. ENR, socially enriched flies.

Synaptic bouton numbers in the ventral lateral neurons (LN_v_s) of the accessory medulla increase when flies are exposed to enriched social conditions versus isolation, whether pre-synaptic or post-synaptic markers are used [46]. We measured the changes in brain morphology under conditions of social enrichment. The lines from both the DGRP and the SIP are derived from wild-caught flies; hence transgenic pre- or post-synaptic markers cannot be used. However, Pigment Dispersing Factor (PDF) itself is a marker for synaptic boutons [35,47,53]. We therefore tested whether PDF positive terminals revealed by immunostaining would exhibit the same property as the transgenic markers after social enrichment in DGRP_373. First, we expressed a pre- as well as a post-synaptic marker in the PDF positive neurons. We used Pdf-GAL4 to drive UAS-Dlg-GFP and UAS-VAMP-GFP in flies, then dissected the brains and co-labelled them with anti-PDF antibody. We observed PDF co-staining with GFP markers in the LN_v_s terminals, consistent with earlier reports (Figure S2, Video S1) [35,47,53]. We then applied social isolation and enrichment treatments for 5 days to DGRP_373 and assayed the flies’ sleep for 3 days after the treatment. On the 9^th^ day, the brains of these flies were dissected and stained with anti-PDF antibody. We counted the numbers of PDF release sites in the accessory medulla after social enrichment and social isolation. We observed the expected pattern of behavior in the DGRP_373 control: daytime sleep increased in socially enriched animals, and the numbers of PDF-positive terminals increased as well (Figure 4A and 4B; Figure 5). Daytime sleep increased from 306.4 ± 15 min in isolated females to 405.8 ± 11.8 min in socially enriched females. The corresponding number of positive terminals were 385 ± 13.4 in isolated females and 571.7 ± 25.6 in females exposed to social conditions (*p* < 0.0001), a 48.4% increase over the isolated condition. Day sleep in males increased from 445.3 ± 11.4 min when isolated to 543.9 ± 9.6 min when socially enriched. The isolated males of DGRP_373 had 345 ± 12.4 PDF positive terminals on average, while socially enriched flies had 577.1 ± 15.1 sites (*p* < 0.0001), a 67.3% increase over the isolated condition. Thus, exposure to enriched social conditions increased daytime sleep and altered the morphology of LN_v_ neuronal projections in DGRP_373 as anticipated.

We next tested changes in sleep and morphology in a representative long sleeping SIP line, SIP_L1_1, and a short sleeping SIP line, SIP_S2_7, using the same paradigm as indicated above. Females of SIP_L1_1 did not exhibit an increase in day sleep after social enrichment, while males responded to social exposure with an increase in day sleep from 513.6 ± 8.6 min to 585.2 ± 6.1 min (*p* < 0.0001) (Figure 4C). Unlike the moderate-sleeping DGRP_373, we found no differences in the number of PDF positive terminals between the socially enriched and the socially isolated condition for males or females of SIP_L1_1 (Figure 4D; Figure 5). Positive terminals in females increased slightly from 449.5 ± 30.6 sites to 515.0 ± 24.8 sites, as did males: 497.6 ± 13.3 sites for isolated males versus 544.4 ± 20.5 sites for enriched males. Like SIP_L1_1 females, SIP_S2_7 females did not respond to the social enrichment paradigm with increased day sleep; however, SIP_S2_7 males did, increasing day sleep from 165.7 ± 15.2 to 311.8 ± 13.2 min (Figure 4E). When we examined the PDF positive terminals in the brains of these flies, we did not observe any significant differences between socially isolated or enriched flies (Figure 4F; Figure 5). Thus, the expected sleep and morphological changes due to social enrichment did not occur in these two lines.

Previous work demonstrated that older flies do not respond to social enrichment with increased sleep and increased LN_v_ punctae [45]. Eleven-day old flies responded robustly with increased day sleep and morphological changes, while 26-day old flies did not [45]. We wondered whether morphological changes in the SIP_L1_1 and SIP_S2_7 due to social enrichment might be sensitive to time, resulting in a more rapid decay of the effect of social enrichment. We thus examined the brains of flies that had undergone the social isolation and enrichment treatments immediately after 5 days of social enrichment. We observed significant changes in PDF positive terminals in the expected direction for SIP_L1_1 females. These flies had an increase of 132.7 (33.8%) PDF positive terminals on average when socially enriched (*p* = 0.0128). PDF positive terminals for SIP_L1_1 males were slightly increased in number for socially enriched flies versus isolated flies—9.8 sites on average (*p* = ns). The average number of PDF positive terminals in SIP_S2_7 females actually decreased under socially enriched conditions, however, from 462.8 ± 30.5 under socially isolated conditions to 318.7 ± 21.9 under social enrichment (*p* = 0.0004), a decrease of 31.1%. In contrast, SIP_S2_7 males had slightly increased numbers of PDF positive terminals when isolated (553.5 ± 38.8 versus 527.7 ± 23.9), though the increase was not significant. Thus, the morphological changes we observed, like aversive phototaxis assay responses, suggest that some defect in neural plasticity may exist in extreme long and short sleeping flies.

### 2.5. Circadian Rhythms in SIP Representative Lines

**Figure 6:**
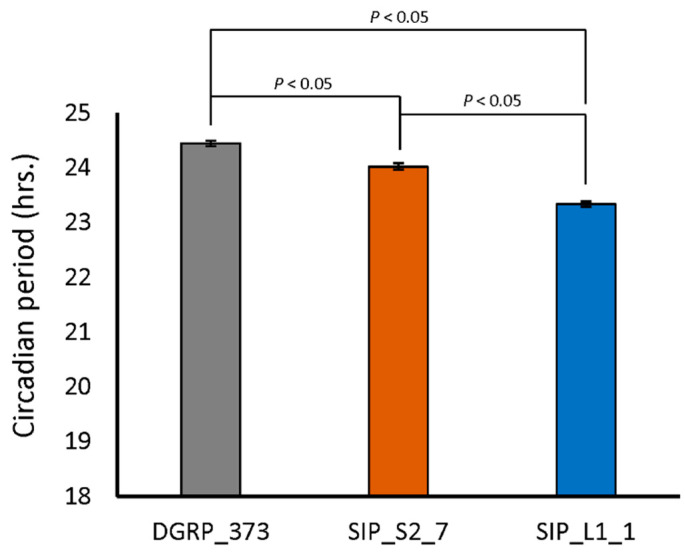
Circadian period in representative lines of the SIP.

Recent evidence has been accumulating that biological clocks, like sleep, have a role in synaptic plasticity (reviewed in [54]). We therefore examined the circadian behavior of DGRP_373, SIP_L1_1, and SIP_S2_7. Both SIP_S2_7 and SIP_L1_1 had slightly, though significantly, lower circadian periods than DGRP_373 (Figure 6). DGRP_373 had a circadian period of 24.5 ± 0.05 hrs., while SIP_S2_7 and SIP_L1_1 had circadian periods of 24.0 ± 0.06 h and 23.3 ± 0.05 h, respectively. Interestingly, 47.5% of the flies of line SIP_S2_7 were arrhythmic, while only 5% of the flies of line DGRP_373 and 0% of the flies of SIP_L1_1 were arrhythmic. This observation uncovers the possibility that the anomalies we observed in memory acquisition, sleep behavior, and morphology are linked to the higher percentage of arrhythmicity in the short sleepers.

## 3. Discussion

Our results have potentially important implications for the relationship between sleep and learning and memory. First, both long and short sleeping lines of the SIP were uniformly deficient in their performance on the aversive phototaxis suppression assay. Neither group’s short-term memory could match the moderate-sleeping control. Moderate-sleeping DGRP_373 had better performance than widely-used laboratory strains [22,55], but the performance scores of the long and short sleeping lines of the SIP were similar to that of *lio*, *pastrel*, *dunce*, and *rutabaga* mutants [23] and flies artificially selected for insomnia-like symptoms [56]. This inability to form short-term memories was due to a defect in the acquisition of memory rather than its retention. Some lines did not show any improvement in their ability to choose the darkened chamber over time. Other lines did improve with time, but at a much slower rate than the moderate-sleeping control. One possibility is that the poor performance is confined to the aversive phototaxis assay. The flies could potentially have better performance on other types of learning and memory assays, such as the heat box assay for short-term memory [24], or the olfactory learning [26] and courtship learning and memory assay for long-term memory [27]. However, we did not find any physiological defects that might have interfered with the aversive phototaxis test. All flies were tested initially for phototaxis, and only phototactic flies were trained and tested. We found that 17.2% of the flies were not phototactic, which is similar to the number found in previous studies [22]. The SIP also responded normally to both water and quinine, demonstrating the expected aversion to quinine. Future research will examine the response of the SIP to other types of learning and memory assays.

Nor did the SIP respond as expected to the social exposure paradigm when their sleep-wake behavior was examined. While most males increased their day sleep in response to social enrichment as predicted, females were less likely to respond with increased day sleep, and some lines actually had decreased day sleep in response to social enrichment. These differences were not due to compensatory increases in night sleep, though in some cases there was an increase in the consolidation of sleep as has been observed previously [45]. In addition, both a long and a short sleeper line lacked the expected changes in sleep and brain morphology after social enrichment, though the trend was an increase in the numbers of PDF release sites in the accessory medulla after social enrichment. Examination of the brains immediately after 5 days of social enrichment revealed a large increase in release sites in females of the long sleeper line, SIP_L1_1 but a decrease in the short-sleeping females of SIP_S2_7. The decrease in the number of release sites in the short sleepers was not expected. However, a previous study demonstrated that Kenyon cells in flies that had been awake for a long time lost their ability to respond to a stimulus [57]. One possibility, therefore, is that the SIP_S2_7 females, who averaged 204.9 ± 35.7 min of sleep in a 24-h period, were unable to respond to the additional stimulus. A second possibility is that the decrease in the number of release sites may involve the circadian clock. Almost half of the SIP_S2_7 short sleepers were arrhythmic in their sleep and activity patterns. A polymorphic variant in an intron of *shaggy* (*sgg*) segregates between long and short sleeper lines of the SIP [18,51]. Decreased *sgg* expression lengthens circadian period, while increased expression shortens it [58]. Interestingly, *sgg* has been implicated in olfactory habituation, though the habituation phenotype was independent of the circadian phenotype [59]. It would be interesting to determine whether this polymorphism affects the morphological response to social enrichment in the short sleepers.

Some caveats should be made with respect to the morphological measurements. First, we counted the numbers of PDF release sites in the accessory medulla of the optic lobes, a paradigm that has been previously established [45,46]. However, our measure could potentially miss more subtle differences due to changes in release site volume or size [47]. Second, we examined the response of a single type of morphological change in the brain. The Sleep Inbred Panel’s ancestry originates from ten lines of the Drosophila Genetic Reference Panel that were intercrossed to create an outbred population [18]. Three of the ancestral DGRP lines we used to create the outbred population had gross morphological defects in the β lobes of their mushroom bodies in 5–15% of the flies examined [60]. Specifically, some of the flies had missing or fused β lobes [60]. The mushroom bodies (MBs) are critical for regulating both sleep and learning and memory in *Drosophila*. Previous reports have shown that the sleep duration is affected by both cyclic-AMP-dependent protein kinase (PKA) and dopaminergic activity in the MBs [61,62]. Blocking synaptic transmission in the MBs reduced sleep duration [63]. Similarly, blocking synaptic transmission within the MBs also prevented the retrieval of short-term memories after training using the aversive phototaxis assay [23]. In addition, the MBs are implicated in short-term and long-term memory consolidation [27]. Therefore, mushroom body defects, if present in the SIP, could potentially explain the deficiencies in learning that we observed, a possibility yet to be examined in the SIP under the social isolation and enrichment paradigm.

Interestingly, both very long and very short sleep duration is associated with poor cognitive functioning in humans. In three prospective studies, an inverted U-shaped distribution was observed between sleep duration and multiple measures of cognition [64,65,66]; that is, people with very long or very short sleep duration had poorer performance on cognitive tests than those with moderate sleep duration. This was also the case for two cross-sectional studies [67,68]. However, in five additional studies, lower scores on cognitive tests were associated with longer sleep duration only [69,70,71,72,73]. An additional study reported more cases of dementia among those with long sleep duration than moderate or short sleep [74]. In contrast to these findings, four longitudinal studies found cognitive impairment among those with short sleep duration [75,76,77]. In a meta-analysis of sleep studies, Lo et al. estimated higher odds for poor performance on tasks such as executive function, verbal memory, and working memory capacity in those with long sleep duration as compared to those with moderate sleep duration [78]. Yet those with short sleep duration fared no better and performed just as poorly on the same types of tasks [78]. Although an inverted U-shaped distribution is not evident in flies, there is a parallel between the relationship of sleep duration and cognitive impairment in humans and the observations we made in short and long sleeping flies of the SIP. Fragmentation of daily rhythms was also associated with decreased cognitive performance in humans, apart from sleep duration [79,80,81]. Thus, the SIP could be used as a model to further explore the genetic underpinnings of the complex relationship between memory acquisition, sleep duration, and circadian rhythms.

## 4. Materials and Methods

### 4.1. Fly Stocks

We tested flies of the 39-line Sleep Inbred Panel (SIP) [18], a panel of long and short sleeping wildtype lines. We used a line from the Drosophila Genetic Reference Panel [48,49], DGRP_373, as a control. We chose this line as a control because it had the closest median day and night sleep (364.4 min and 567.9 min, respectively) to the median day and night sleep of all the DGRP lines (370.1 min and 568.3 min, respectively) [10]. The UAS-dlgWT-GFP flies were obtained from B. Lu (Stanford University) and UAS-VAMP-GFP flies were obtained from A. DiAntonio (Washington University in St. Louis). Pdf-Gal4 flies (stock no. 6899) were obtained from the Bloomington Drosophila Stock Center (Indiana University).

### 4.2. Aversive Phototaxis Assay

We tested flies using the aversive phototaxis assay, an assay that exploits the natural phototactic behavior in flies by training them to avoid a lighted chamber with an aversive stimulus [22,23]. A T-maze was three-dimensionally printed using the schematic in Figure S3A on a uPrint SE Plus 3D printer (Stratasys, Eden Prairie, MN, USA) with acrylonitrile butadiene styrene plastic. The width of the T-maze alley was 2 mm and the height was 1.4 mm. A ceiling was constructed using two layers of optically clear plastic (School Smart, Appleton, WI, USA) using general purpose super glue (Scotch, St. Paul, MN, USA) with a red filter (Rosco, Stamford, CT, USA) between the two layers as *Drosophila* are nearly blind to red light [22]. Holes were cut in the ceiling at the two ends of the T-maze. A gray opaque PVC disc (McMaster-Carr, Princeton, NJ, USA) was glued to each opening of the ceiling. The inside diameter of the disc measured 6.2 mm and the outside diameter measured 13 mm with an average thickness of 1.5 mm. An opaque gray PEEK sleeve bearing (McMaster-Carr, Princeton, NJ, USA) was inserted on each of the opaque gray discs to create receptacles for the vials (12.7 mm inside diameter; 15.1 mm outside diameter; 12.7 mm long). A 3 mL polystyrene vial (Globe Scientific, Paramus, NJ, USA) containing one sheet of 55 mm diameter circular filter paper (Whatman, Maidstone, UK) was placed inside each of the sleeve bearings to create two different choices for the fly. One vial was covered in aluminum foil (Reynold’s Wrap, Lake Forest, IL, USA) to create a darkened vial and the other vial was illuminated by a gooseneck light (Schott-Fostec, Elmsford, NY, USA) to create a lighted vial. Laboratory tape (Daigger, Vernon Hills, IL, USA) was used to hold the ceiling in place during experimentation. Figure S3B shows the experimental setup of the maze.

The flies were raised on standard food (http://flystocks.bio.indiana.edu/Fly_Work/media-recipes/bloomfood.htm), in constant conditions (25.0 °C temperature, 50–60% humidity, and 12 h:12 h light:dark cycle). To create an aversive stimulus, 4.96 g of quinine hydrochloride (Sigma Aldrich, CAS Number: 6119-47-7, St. Louis, MO) was dissolved in 125 mL of distilled water to make a 0.1 M quinine hydrochloride solution. The experiments were conducted in a 21–25 °C room with the only light source being the gooseneck light for the lighted vial. Newly eclosed flies were collected under CO_2_ anesthesia and placed individually in collection vials until they were 4–7 days old. Assays for one representative long sleeping line and one representative short sleeping line (SIP_L1_1 and SIP_S2_7, respectively) along with DGRP_373 revealed that holding the flies individually or in the socially exposed condition prior to the assay did not affect their ability to learn (Figure S4). Trials began at ZT0, the lights on time in the incubator. Flies were randomized according to genotype and sex, and trials were conducted until 4 flies completed the experiment for that day, usually between ZT2 and ZT3. Five flies per sex per line were assayed (400 flies total).

For each fly, the first two trials were a test for natural phototaxis. Each fly was inserted into the maze where it could make a choice between the lighted and darkened vial. Once the fly reached a vial, the trial ended, and the fly was captured back into the collection vial. If the fly chose the lighted vial, the vials were switched to have the lighted vial on the opposite side from the first trial. If the fly chose the darkened vial at any point during the first two trials, we noted that it failed to exhibit phototaxis, and the fly did not continue through the learning and memory assay. We found that 17.2% of the flies did not exhibit phototaxis behavior in the first two trials. If the fly chose the lighted vial for both trials, it was said to exhibit phototaxis and the fly continued the experiment. For the next 16 trials, the filter paper was soaked with 350 µL of 0.1 M quinine hydrochloride solution to act as an aversive stimulus. The filter paper in the darkened vial was left dry. Each fly was tested for four blocks of four trials using a double alternating pattern (LLRR LLRR or RRLL RRLL), which allowed the lighted vial to be on the right side for half of the trials and the left side for the other half of the trials. We recorded the fly’s choice of vial for each trial. After the fly completed 16 trials, the vials were discarded, and the ceiling and maze were washed with distilled water and dried. We used the results of the last block of four trials to calculate the fly’s performance score [23] which ranged from 0 to 4.

### 4.3. Proboscis Extension Reflex Assay

Four to seven-day old flies were anesthetized using CO_2_ and placed in an empty culture vial for 1.5 h. After 1.5 h in the empty vial, flies were mounted to a glass side using double sided tape. Using a paintbrush, 20 flies per line/sex (1600 total) were positioned on the slide so that their wings were stuck to the tape and their proboscis could easily be seen using a microscope. After allowing the flies to recover from anesthesia, the glass slide was placed under a light microscope (Leica, Wetzlar, Germany). Using a 0.5–10 μL pipette (Eppendorf, Hamburg, Germany), a drop of water was continually rubbed against the legs and abdomen of the first 10 flies on the slide for 5 s. If the fly’s proboscis extended any time during the 5 s, the fly was said to have the proboscis extension reflex. The same procedure was done for the next 10 flies using 0.1 M quinine hydrochloride (Sigma Aldrich, CAS Number: 6119-47-7, St. Louis, MO, USA) solution. A proboscis extension reflex indicated an appetitive stimulus and no extension reflex indicated an aversive stimulus. The percentage of flies with a proboscis reflex was then compared between water and quinine hydrochloride solution. Each line of the SIP was assayed for proboscis extension reflex as well as the DGRP_373 control. The experiment was conducted in 10 blocks of 4 lines. 10 males and females of each line were assayed. The average response to quinine for each line was computed as the difference between mean response to quinine and the mean response to water.

### 4.4. Sleep Measurements After Social Isolation or Social Enrichment

Fly cultures were seeded with 5 males and 5 females per vial and maintained on standard food (https://bdsc.indiana.edu/information/recipes/bloomfood.html) under standard conditions (25 °C, 60% humidity, 12 h:12 h light:dark cycle). Virgin males and females were collected from cultures and separated into two treatments: socially isolated and socially enriched. Isolated flies were housed as one fly per culture vial; enriched flies were housed as 30 flies to a same-sex vial. After the flies were housed for 5 days in either isolated or enriched conditions, they were transferred to DAM5 monitors (Trikinetics, Waltham, MA) and sleep and activity was monitored for 3 days [20,21]. Flies were placed into 5 mm × 76.2 mm tubes, which were inserted into the monitors. Each monitor uses infrared beams that bisect the tubes to count the number of times per minute that the fly walks back and forth along the tube. Five minutes without an activity count is defined as sleep [82]. Sixteen flies were set up for each SIP line/DGRP control, sex, and treatment (2496 flies total). Thirty-two flies were set up for DGRP_373, SIP_L1_1, and SIP_S2_7 per sex and treatment (384 flies total). Flies were visually inspected after sleep and activity monitoring; data from flies that died during the experiment was not used. Using a C# program, Sleep Analysis 6.1 (R. Sean Barnes), we calculated the sleep duration, numbers of sleep bouts, and average sleep bout length for both day and night; the sleep latency, which is the number of minutes before the first sleep bout during the night; and the waking activity, which is the number of activity counts per minute spent awake.

We conducted a pilot experiment to test the effects of social enrichment using an outbred population of flies created in a previous study [51]. This population was replicate 1 of a control population with moderate average sleep duration (495.1 ± 11.71 min) [51]. Using the procedure above, we measured sleep for flies housed at 1 fly per same-sex vial, 5 flies per same-sex vial, 20 flies per same-sex vial, and 30 flies per same-sex vial. Day sleep increased linearly with increased numbers of flies per vial, as reported previously by other investigators (Figure S5A) [30]. We harvested 3 brains per housing treatment/sex and compared the numbers of PDF release sites. Flies with greater social enrichment had greater numbers of release sites (Figure S5B), as anticipated.

### 4.5. Immunostaining Protocol

We stained the brains of DGRP_373, SIP_L1_1, and SIP_S2_7 flies at two time points. For the first time point, flies were subjected to either isolation or social exposure treatment for 5 days, sleep was measured for 3 days, and on the 9^th^ day 24–32 brains per line/sex/treatment were harvested (338 brains total). For the second time point, SIP_L1_1 and SIP_S2_7 flies were subjected to either isolation or social exposure treatment for 5 days and then 16–20 brains per line/sex/treatment were harvested (150 brains total). Brains were immunostained for Pigment-dispersing factor (PDF) using the following protocol. Flies were rinsed in 100% ethanol, followed by a wash in 1X PBS (Phosphate Buffer Saline, Quality Biologicals, Inc., Gaithersburg, MD, USA). Flies were then transferred into freshly made 4% PFA (Sigma-Aldrich, St. Louis, MO, USA) in order to fix the brain tissue. The head case (cuticle) was opened in the 4% PFA by removing the body and eyes around the optic lobes. Once the brain was exposed, they were moved into a separate well within the dissection dish in 4% PFA and fixed for 20-25 min. After the brains were fixed, they were cleaned in 1X PBS-T (tween 0.2%) by removing remaining tissues. After all dissections were complete, the brains were washed three times in 1X PBS-T at room temperature on a nutator/shaker for 30 min, followed by incubation in blocking solution (Normal Donkey Serum, Jackson Labs, Bar Harbor, ME) for another 30 min. Afterwards, the brains were incubated overnight with primary antibodies (rabbit anti-PDF; 1:500; a gift from Amita Sehgal, University of Pennsylvania) at 4 °C. The next day, the primary antibody solution was carefully removed from each individual brain without disturbing them and then washed three times with 1X PBS-T for 30 min at room temperature. The brains were incubated with secondary antibodies [(anti-mouse Alexa 633, 1:500 and anti-rabbit Alexa 488, 1:500, Thermo Fisher Scientific, Waltham, MA, USA)] at room temperature for 2 h wrapped in aluminum foil, followed by three washes with 1X PBS-T for 30 min. Wash solutions were carefully removed and the brains were mounted on a glass slide (75 × 75 × 1 mm Azer Scientific, Morgantown, PA) with Vecta Shield (Vector Laboratories, Burlingame, CA, USA) and coverslip (Vector Laboratories, Burlingame, CA, USA) and sealed with nail polish.

### 4.6. Confocal Imaging and Quantification of PDF Release Sites

Brains were imaged with a laser scanning confocal microscope (SP5 Leica). Confocal stacks were acquired with a 0.3 μm slice thickness using identical microscope settings. Imaris software (Bitplane, Oxford Instruments, Concord, MA, USA) was used to quantify the PDF release sites in the accessory medulla of both brain hemispheres in socially isolated and socially enriched flies. Specifically, the spots application module was used to automatically acquire and count the PDF release sites. The size of individual spots was kept constant at 0.3 microns for every experiment.

### 4.7. Co-Staining of PDF with Pre-Synaptic and Post-Synaptic Markers

Since PDF is a neuropeptide and not a synaptic marker, its presence within synaptic zones is unknown. To determine whether PDF is released in the synaptic zones, we used transgenic flies expressing GFP fused with pre or post-synaptic marker proteins to determine the effect of social enrichment on sleep and synaptic plasticity. Co-localization of PDF in the synaptic boutons was determined by co-staining the fly brains with mouse anti-PDF (C7, 1:500, DSHB, University of Iowa) and rabbit anti-GFP (1:1000, Abcam, Cambridge, MA, USA) expressing UAS-DLG-GFP (pre-synaptic marker) and UAS-VAMP-GFP (post-synaptic marker) under the influence of PDF-GAL4 driver [46].

### 4.8. Circadian Rhythm Measurements

We measured the circadian behavior of DGRP_373, SIP_L1_1, and SIP_S2_7. Fly cultures were set up as for the sleep measurements in Section 4.4 above, with the following differences. Virgin males and females were collected and housed at 20 flies to a same-sex vial for five days, then transferred to DAM5 monitors. Thirty-two flies per sex per line were measured. Monitor tubes contained a 5% sucrose, 1.5% agar food, in lieu of the flies’ normal food in order to avoid drying of the food. Normal sleep and activity patterns were first recorded for 3 days in LD. The flies were then switched to constant darkness, and sleep and activity patterns were measured for 14 days. We used Clocklab (Actimetrics, Wilmette, IL, USA) to calculate the circadian period of the flies and to determine whether their activity patterns were rhythmic or arrhythmic.

## Supplementary Materials

The following are available online at https://www.mdpi.com/2624-5175/1/4/36/s1, Figure S1: Correlations between sleep duration and aversive phototaxis score; Figure S2: Co-staining of PDF-Dlg-GFP and anti-PDF reveals PDF is released in the pre-synaptic neurons; Figure S3: Aversive phototaxis suppression apparatus; Figure S4: Learning is independent of prior social exposure; Figure S5: Pilot study of the effects of social enrichment; Table S1: SIP and DGRP_373 line means for the Aversive Phototaxis Assay and Proboscis Extension Reflex to water and quinine; Table S2: Mean sleep parameters for isolated and socially enriched flies of the SIP; Table S3: ANOVA results showing the effect of social enrichment on sleep; and Video S1: Co-staining of PDF-Dlg-GFP and anti-PDF reveals PDF is released in the pre-synaptic neurons.

## Abbreviations

SIP	Sleep Inbred Panel
LNv	Ventral lateral neuron
