# Supplementary material for: Short-Term Memory Deficits in the SLEEP Inbred Panel
Source: Clocks Sleep. 2019 Oct 28;1(4):471–88. doi: 10.3390/clockssleep1040036 (PMC7318870; doi:10.3390/clockssleep1040036)
Supplement: Supplementary file 1 [file clockssleep-01-00036-s001.zip › Supplementary Figures S1-S5.pdf]

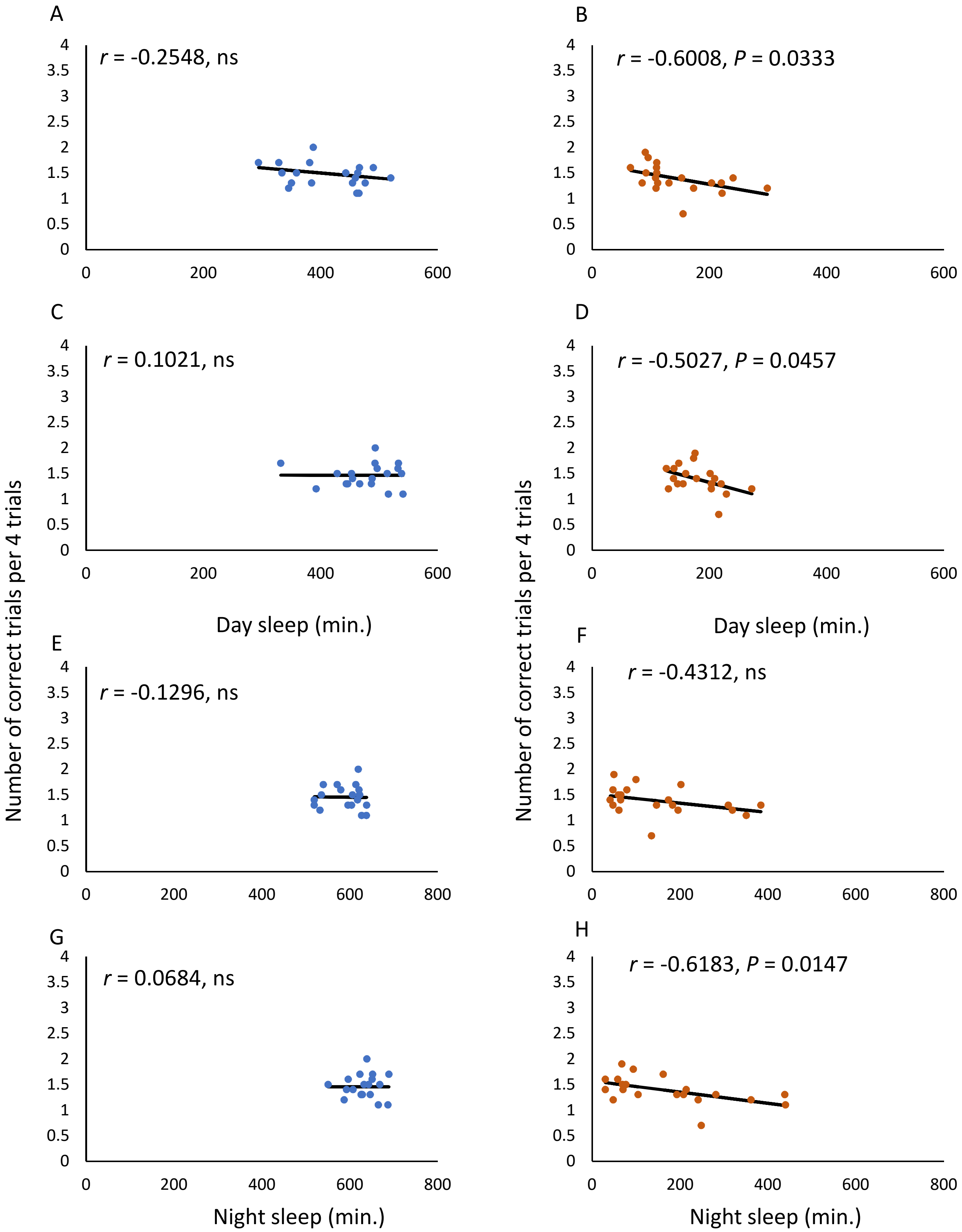

Figure S1. Correlations between sleep duration and aversive phototaxis performance score. (a-d) Spearman correlation between aversive phototaxis score and day sleep for (a) isolated long sleepers; (b) isolated short sleepers; (c) enriched long sleepers; (d) enriched short sleepers. (e-h) Spearman correlation between aversive phototaxis score and night sleep for (e) isolated long sleepers; (f) isolated short sleepers; (g) enriched long sleepers; (h) enriched short sleepers.

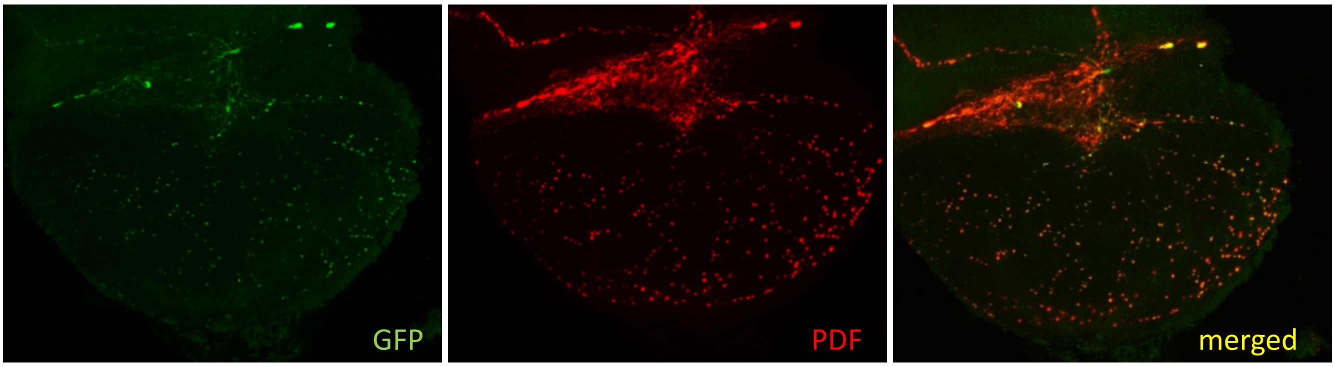

Figure S2. Co-staining of PDF-Dlg-GFP and anti-PDF reveals PDF is released in the pre-synaptic neurons.

A

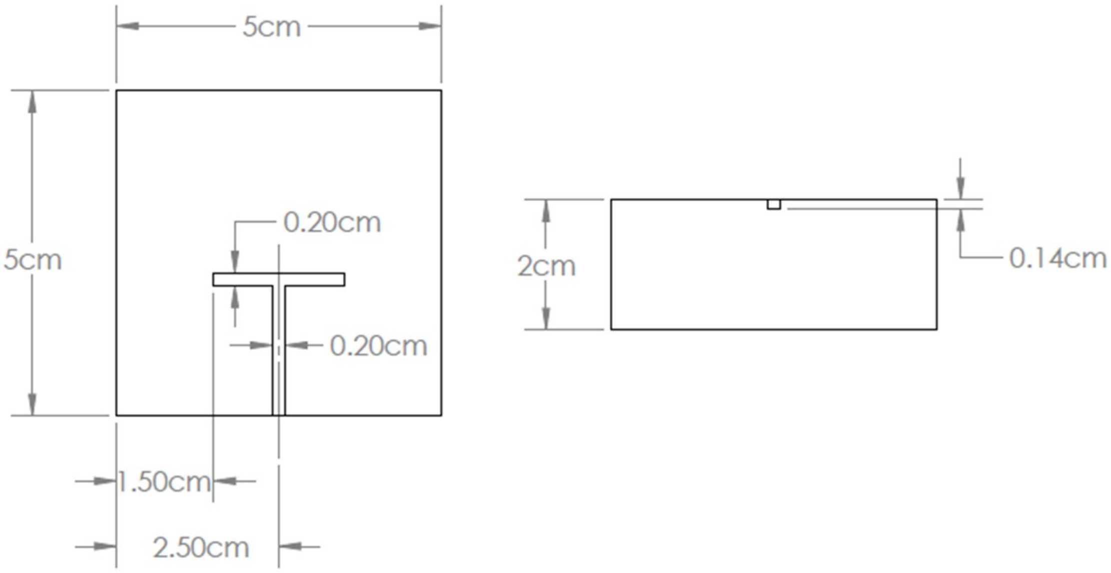

B

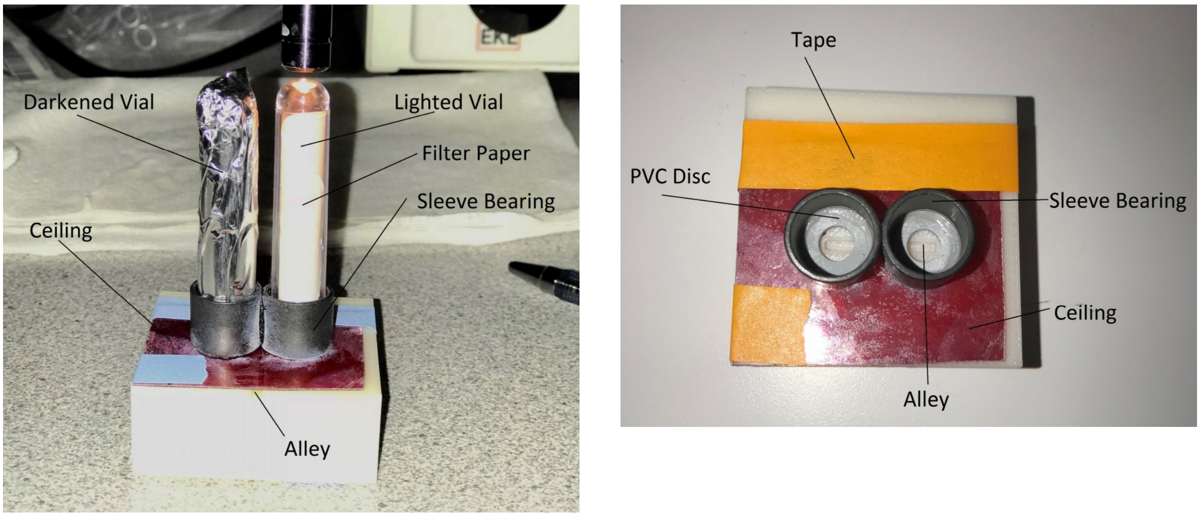

Figure S3. Aversive phototaxis suppression apparatus. (a) Schematic for T maze; (b) experimental setup.

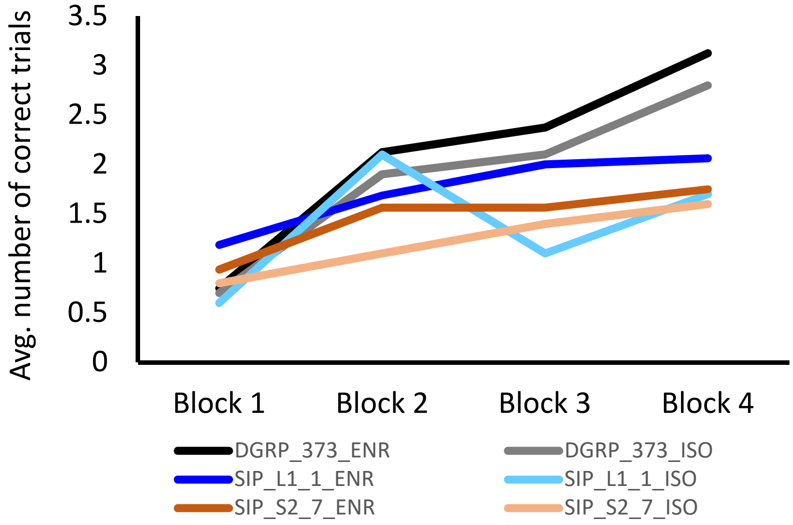

Figure S4. Learning is independent of prior social exposure. The graph shows the average number of correct trials for each block. No significant differences in learning were found for enriched versus isolated flies.

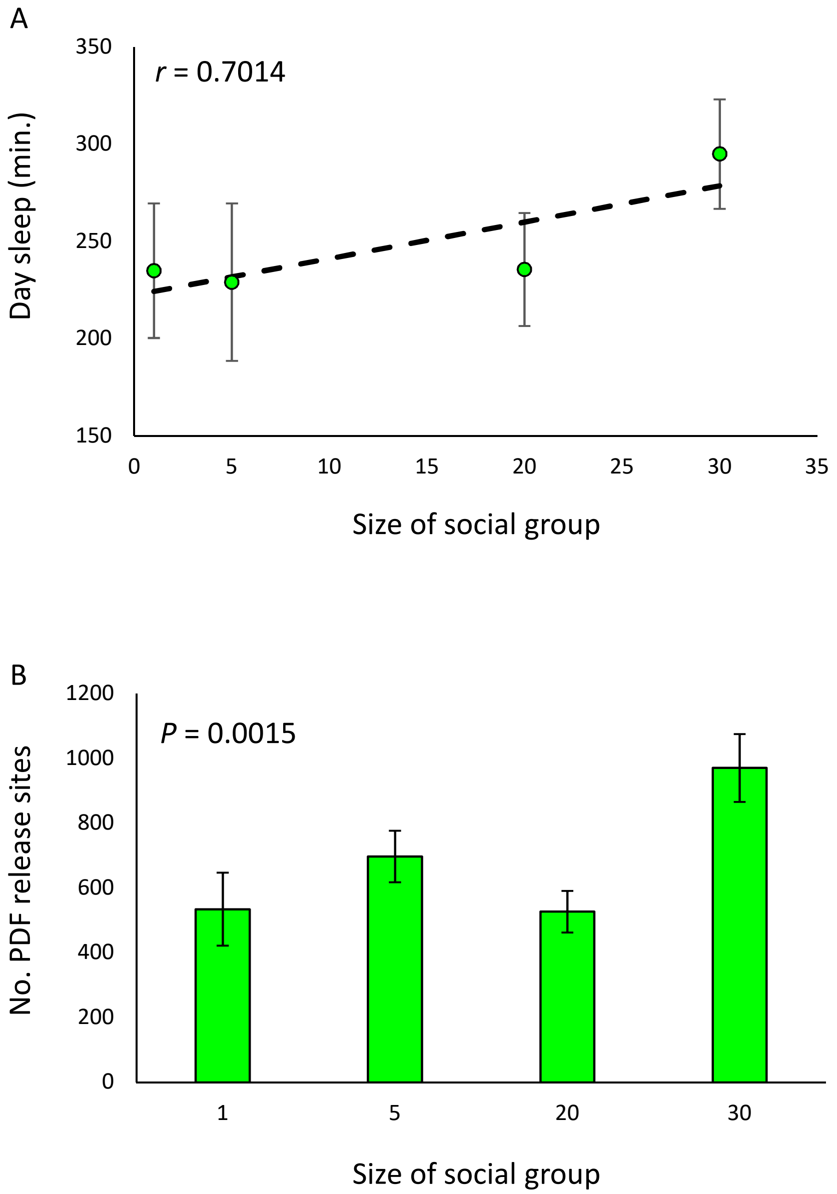

Figure S5. Pilot study of the effects of social enrichment. (a) Relationship between day sleep and social group size. The dashed line shows the linear fit. (b) Relationship between number of PDF release sites and social group size.
